# White matter neuron biology and neuropathology in schizophrenia

**DOI:** 10.1038/s41537-019-0078-8

**Published:** 2019-07-08

**Authors:** Ryan J. Duchatel, Cynthia Shannon Weickert, Paul A. Tooney

**Affiliations:** 1 School of Biomedical Sciences and Pharmacy, Faculty of Health and Medicine, University of Newcastle, Callaghan, NSW 2308 Australia; 2 Priority Centre for Brain and Mental Health Research and Hunter Medical Research Institute, University of Newcastle, Callaghan, NSW 2308 Australia; 3 Schizophrenia Research Laboratory, Neuroscience Research Australia, Randwick, NSW 2031 Australia; 4 School of Psychiatry, Faculty of Medicine, University of New South Wales, Sydney, NSW 2052 Australia; 5 Department of Neuroscience & Physiology, Upstate Medical University, Syracuse, New York, 13210 USA

**Keywords:** Schizophrenia, Schizophrenia, Cellular neuroscience

## Abstract

Schizophrenia is considered a neurodevelopmental disorder as it often manifests before full brain maturation and is also a cerebral cortical disorder where deficits in GABAergic interneurons are prominent. Whilst most neurons are located in cortical and subcortical grey matter regions, a smaller population of neurons reside in white matter tracts of the primate and to a lesser extent, the rodent brain, subjacent to the cortex. These interstitial white matter neurons (IWMNs) have been identified with general markers for neurons [e.g., neuronal nuclear antigen (NeuN)] and with specific markers for neuronal subtypes such as GABAergic neurons. Studies of IWMNs in schizophrenia have primarily focused on their density underneath cortical areas known to be affected in schizophrenia such as the dorsolateral prefrontal cortex. Most of these studies of postmortem brains have identified increased NeuN+ and GABAergic IWMN density in people with schizophrenia compared to healthy controls. Whether IWMNs are involved in the pathogenesis of schizophrenia or if they are increased because of the cortical pathology in schizophrenia is unknown. We also do not understand how increased IWMN might contribute to brain dysfunction in the disorder. Here we review the literature on IWMN pathology in schizophrenia. We provide insight into the postulated functional significance of these neurons including how they may contribute to the pathophysiology of schizophrenia.

## Introduction

This review begins by discussing the changes in the structure of the brain’s cortical grey matter in schizophrenia at a whole brain and cellular level, highlighting the robust changes reported in cortical interneurons. It points out that structural and cellular changes are also observed in the white matter of the brain in people with schizophrenia. It focuses on the studies that have shown increased density of neurons, in the white matter below the cortex in cases with schizophrenia and proposes mechanisms by which this change might occur.

## Grey matter pathology in schizophrenia

Research into the neuropathology of schizophrenia has been guided by brain imaging studies which have identified many regional differences in the human grey matter that has widespread alterations including decreased volumes of cortical grey matter in the dorsolateral prefrontal cortex (DLPFC), cingulate gyrus, medial temporal lobe and superior temporal gyrus.^1^ Cortical thinning in schizophrenia was confirmed in a recent meta-analysis by the ENIGMA (Enhancing Neuro Imaging Genetics through Meta Analysis) schizophrenia working group, in one of the largest sample sizes to date (4475 schizophrenia, 5098 controls).^2^ Interestingly whilst the ENIGMA study showed the thinning is widespread across cortical regions, it was most prominent in frontal and temporal brain regions.^2^

At a cellular level, deficits in interneurons containing γ-aminobutyric acid (GABA) termed GABAergic interneurons, have been a widely reported neuropathological finding in the cortex in schizophrenia.^3,4^ Interneurons are vital for maintaining a balance of excitatory signalling within the brain. Interneurons have traditionally been identified by their expression of glutamic acid decarboxylase-67 (GAD67), the enzyme that synthesizes GABA and then subtyped by their morphological characteristics, expression of other protein markers (e.g., parvalbumin [PV] and somatostatin [SST])^5^ and more recently single-cell RNA-seq data^6,7^, into about 9 different subtypes. Indeed, one of the most robust findings reported in the schizophrenia literature is a decrease in the expression of GAD67 in interneurons.^4^ The literature consistently reports decreased GAD67 mRNA^8–11^ and protein^12,13^ in 25-35% of interneurons^14,15^ in the DLPFC. GAD67 mRNA reductions are widespread and can be found in many brain regions in people with schizophrenia including the anterior cingulate cortex, superior temporal cortices, striatum and thalamus^16^ mirroring the widespread volume reductions observed by MRI. Decreased GAD67 protein and mRNA has been shown to occur in a variety of interneuron subsets, particularly chandelier and basket neurons that express parvalbumin (PV), a calcium binding protein^17^ and the somatostatin positive neurons that are often bipolar dendrite targeting neurons.^3,18,19^ These changes in interneurons are postulated to affect GABA-mediated inhibition, pyramidal neuron excitation and the generation of gamma oscillations in the cortex which is thought to be dependent on fast spiking PV+ interneurons.^20^ This has been postulated to contribute to the development of cognitive deficits in schizophrenia implicating a role for interneuron pathology in the disorder.^21^ Whilst Lewis, et al.^22^ showed a selective GABA_A_ receptor agonist (MK-0777) was able to improve cognitive impairment in people with schizophrenia, a larger study failed to repeat these findings.^23^ The link between PV+ interneuron pathology and cognitive deficits is likely to be more complex than first thought. There is evidence showing disruption to other rhythmic oscillations of different frequencies such as alpha, beta, delta and theta oscillations in schizophrenia,^24,25^ suggesting more than just PV+ interneurons alone contribute to this abnormal EEG rhythms detected in people with schizophrenia. Furthermore, Wöhr, et al.^26^ studied the behaviour of PV knockout mice and concluded they have a phenotype more typical of autism spectrum disorders (e.g., altered reciprocal social interactions, communication impairments and repetitive/stereotypical behaviours) than other psychiatric illnesses including schizophrenia, but did not focus on other domains of cognition. However, PV gene expression is not lost completely in autism nor schizophrenia, rather it is reduced.^27,28^

## White matter pathology in schizophrenia

Whilst most of the early studies investigated the grey matter, white matter pathology in schizophrenia has been increasing in focus particularly as neuroimaging techniques have developed more detailed ways to map white matter regions.^29^ White matter consists of the axonal projections from neurons in one cortical area to other neurons in another cortical area or to neurons in a subcortical area and forms the basis for connectivity, integration and communication in the brain. White matter also contains myelin, glia, blood vessels, perivascular cells and some neuronal cell bodies. Structural integrity of white matter can be examined indirectly with a diffusion-weighted imaging technique called diffusion tensor imaging (DTI) that allows the investigation of location, orientation, and anisotropy of the brains’ white matter tracts and more recently has grown in popularity as a tool used by schizophrenia researchers.^29^ DTI revealed abnormal morphology and decreased integrity of white matter tracts in the brain in people with schizophrenia.^30,31^ Furthermore, these changes in white matter tracts detected by DTI were present in first-episode schizophrenia, as well as individuals at high risk prior to onset of disease symptoms.^32^ A progressive reduction of frontal lobe white matter volume has also been identified using DTI in schizophrenia patients post diagnosis, and this continued with antipsychotic treatment.^33^ During development, there are long lasting structural changes to the white matter that are thought to be necessary for the generation of cognitive functions,^34^ in particular working memory and reading ability. This is most pronounced during the transition from adolescence to early adulthood,^33^ which overlaps with the time of onset of schizophrenia. Therefore, any alteration to white matter development or damage to white matter structures at this time point may contribute greatly to the development of schizophrenia. The evidence for white matter pathology within schizophrenia obtained from more macroscopic tools suggests that a reassessment of white matter neuropathology at a cellular level is needed to help understand the possible biological underpinnings for the imaging changes reported in schizophrenia. In moving forward, it will be important to determine how functional abnormalities predominantly associated with cortical abnormalities in schizophrenia are related to white matter alterations.

The white matter consists of myelinated fibres of neuronal axons, oligodendrocytes, astrocytes and microglia and these have been studied to varying degrees in post mortem brains from people with schizophrenia. Whilst decreased density of oligodendrocytes in the white matter subjacent to the PFC has been observed,^35^ other studies found no changes.^36–38^ Several studies have investigated astrocytes in the white matter predominantly below the anterior cingulate cortex (ACC) from post mortem brains of people with schizophrenia using glial fibrillary acidic protein (GFAP) at the protein or mRNA level as a marker, but once again consistency is lacking. Williams, et al.^37^ reported a reduction in GFAP+ astrocyte density, Katsel, et al.^39^ reported no change in several astrocyte markers and Webster, et al.^40^ reported increased GFAP mRNA all in the white matter of the ACC in cases with schizophrenia. Differences could be due to differences in techniques used. More recently microglia have been studied with the focus on neuroinflammation (discuss in more detail below). Whilst Fillman, et al.^41^ reported increased density of HLA-DR+ microglia in the white matter under the DLPFC in cases with schizophrenia, Hercher, et al.^38^ reported no changes in IBA-1+ microglia. More recently, attention has also turned to the state of microglial activation using both traditional post mortem tissue studies and newer positron emission tomography studies (for review see Laskaris, et al.^42^). However, the most replicated cellular changes reported in schizophrenia literature relate to the density of neurons in the white matter. As such this review focuses on this evidence to provide some hypotheses about how this might impact on cortical dysfunction in the disorder.

## White matter neurons—neurobiology and neurochemistry

In traditional neuroscience, the grey matter of the cortex was thought to contain all the neuronal cell bodies in the brain. However, a small number of neurons have been found to reside within the white matter spaces of the human brain and whilst not as comprehensively studied, should not be discounted when considering the pathophysiology of schizophrenia. These interstitial white matter neurons (IWMNs) were first described in the human brain by Meynert in 1867 when “solitary” neurons were observed between myelinated nerve fibres of the subcortical white matter.^43^ A similar population of neurons was identified by Cajal, who observed neurons between axon bundles of the trigeminal nerve and the white matter of the human cerebellum.^44^ In addition, many neurons were observed with ascending projections towards the overlying cortex and Cajal was the first to postulate that these white matter neurons may be cells displaced from the grey matter.^45^ However, even since the early discovery of these cells, little progress has been made in understanding their biology, in particular their relevance to disease. In this review, we outline IMWN biology, and focus on their role in the pathogenesis of schizophrenia.

Initial studies of IWMNs focused on their location within the brain and morphological characteristics. IWMNs are a morphologically and neurochemically heterogeneous population of cells. From the earliest drawings of Meynert and Cajal of Golgi stained neurons, it was clear that IWMNs had different morphologies and orientations in the human brain.^46,47^ Since then, immunohistochemistry utilizing nicotinamide-adenine dinucleotide phosphate-diaphorase (NADPH) histochemistry and antibodies to neuronal markers has become the most utilized method for examining IWMN subtypes and their distribution. In adults, IWMNs are more abundant in the superficial white matter close to the grey matter boarder, with numbers declining towards the deep white matter.^48^ The frontal cortices contain the highest IWMN density in the adult human brain, followed by the cingulate cortex, then the visual and temporal cortices.^48,49^ Studies of IWMNs in fetal and adult human brains observed that NADPH-positive (NADPH+) IWMNs have predominantly a fusiform shape with some having pyramidal-like, bipolar or multipolar shapes within the superficial white matter under the temporal and frontal cortices.^48,50,51^ Axons from some NADPH+ IWMNs in the adult brain project into the overlying cortical layers.^50^ Furthermore, axons from some superficial IWMNs projected through layer VI to layer V whereas deep IWMNs projected axons only to Layer VI.^52^ IWMNs also exist in other species such as rodents where they have similar morphological diversity and evidence for connections with the cortex.^52^ This indicates there is variability in the morphological characteristics of IWMNs, as well as their dendrites and axonal projections that in turn may be dependent on their location within the white matter. Excitingly, the fact that projections are sent into the grey matter suggests that IWMNs may be communicating directly with overlying neurons to impact cortical processing.

Studies in humans and animals at various developmental ages have investigated the neurochemistry of IWMNs using antibodies directed at proteins expressed in neurons. Antibodies to neuronal nuclei antigen (NeuN), which is used as a marker of mature neurons, appears to identify the overall population of mature IWMNs.^19,48,53–56^ Other markers appear to detect only a subpopulation of IWMNs. For example, NADPH histochemistry labels a proportion of IWMNs, as do antibodies to microtubule associated protein-2 (MAP2).^49,50^ Some IWMNs contain neuronal nitric oxide synthase (nNOS) suggesting they release nitric oxide as a transmitter. IWMNs are immunoreactive for glutamate and type II Ca^2+^/calmodulin-dependent protein kinase-α indicating some are likely excitatory.^57^ Some researchers believe that IWMNs maybe remnants of the developmental subplate that gives rise to cortical neurons and Clancy, et al.^58^ suggests that 15–25% of these persistent subplate neurons in rodents are GABAergic. Indeed, IWMNs are immunoreactive for GAD,^19^ the calcium binding proteins PV and calretinin (CR),^49^ and the neuropeptides neuropeptide Y (NPY), SST,^56,59^ substance P and cholecystokinin.^60^ These studies suggest IWMNs are morphologically and neurochemically diverse, but are all IWMNs truly mature and integrated into cortical circuitry?

Cajal’s drawings of Golgi labelled sections of early postnatal human and rodent brain provided the first evidence that not only do IWMNs project axons to the cortex, but that cortical neurons such as layer VI pyramidal neurons, project axons or axon co-laterals into the superficial white matter. Just under a century later, Kostovic and Rakic^61^ described both symmetrical (putative GABAergic) and asymmetrical (putative glutamatergic) synapses on the dendrites and soma of IWMNs situated below the monkey and human visual, somatosensory and motor cortices, suggesting that these are axons in contact with IWMNs and are equipped with presynaptic terminals that would enable direct neural communication. García-Marín, et al.^48^ studied the synaptic innervation of the adult human frontal, striate and visual cortex and underlying white matter using immunolabelling for GAT-1 (GABA transporter-1) and vGAT (vesicular GABA transporter) to identify GABAergic terminals and vGlut-1 (vesicular glutamate transporter) to identify glutamatergic terminals. The cortical layers had abundant labelling for GABAergic and glutamatergic terminals and whilst they were also evident in the white matter, their density was significantly less.^48^ This anatomical data shows that IWMNs receive synaptic inputs from either other IWMNs, or neurons in the overlying cortex or possibly from subcortical structures, suggesting they are integrated into neural circuits. However, the functional consequences of these putative synapses on IWMNs in frontal brain regions remain a mystery.

Indeed, very few studies have investigated the electrophysiology of IWMNs due to their sparse distribution amongst dense fibre tracts of the white matter making it difficult to locate and record from IWMNs. Clancy, et al.^52^ utilized post recording analysis of biocytin filled neurons in the white matter underneath the visual cortex from postnatal day (P)4-35 rats. Most IWMNs had short-duration action potentials with fast after-hyperpolarisations and virtually no spike-frequency adaptations.^52^ Interestingly, this is very similar to recordings from neurons in cortical layer I,^52^ suggesting IWMNs are electrically active. A study of the visual cortex in young P10-20 rats recorded from subplate neurons (located between the base of layer VI and before the distinct fibres of the white matter) and IWMNs (identified as deeper amongst the white matter fibres).^62^ IWMNs and subplate neurons had similar intrinsic properties and threshold for spike initiation, as well as action potential kinetics.^62^ IWMNs were, however, more depolarized at rest than subplate cells.^62^ Friedlander and Torres-Reveron^60^ in a follow-up study used paired whole-cell patch-clamp recordings from an IWMN and a neuron in cortical layer VI of the rat visual cortex at P20 and suggested that IWMNs received both excitatory and inhibitory synaptic inputs, potentially participating in local synaptic networks. One of the difficulties faced by these earlier researchers was the uncertainty around simply identifying IWMNs to record from using normal light microscopy, plus not knowing what type of neuron the recordings were made from. To solve this problem, Engelhardt, et al.^63^ used young transgenic mice (P10-P30) where the serotonin 5HT_3_ receptor gene was tagged with enhanced green fluorescent protein (eGFP) in the only electrophysiology study of a subset of GABAergic IWMNs. 5HT_3_ eGFP IWMNs underneath the motor cortex at the level of the hippocampus had firing patterns typical of interneurons, receiving excitatory and inhibitory synaptic inputs from cortical and subcortical structures.^63^ In only four paired recordings, they showed that 5HT_3_-positive IWMNs inhibited cortical neurons.^63^ These studies provide tantalizing evidence that IWMNs are indeed functionally integrated into cortical circuitry and are preforming a suppressive role, but they also raise the interesting question: Could this inhibitory function of IWMNs impact on normal brain function or disease states? However, like most aspects of biology, this question is more complex than first thought. Indeed, later in this review (Section 1.4), we also propose and provide some evidence to suggest that a proportion of IWMNs may not be stationary, but that they could be migrating towards the cortex. As such, these connections between IWMNs and cortical/subcortical neurons may be transient. Further research using eGFP tagged neurons in mice could help to unravel which types of IWMNs make connections with the cortex or subcortical regions, how stable these connections are and whether these connections influence normal brain function and disease pathogenesis.

## White matter neurons in schizophrenia

One of the most robust white matter pathologies in people with schizophrenia, is an increased density of IWMNs directly underneath particular cortical regions implicated in the disorder. To date fourteen studies have examined the density of IWMNs in the postmortem brains of schizophrenia subjects. Eight out of the fourteen studies have shown an increase in the density of neurons in the superficial white matter below the cortex,^19,53–56,64–66^ with three studies showing increases in the deep white matter.^67–69^ In contrast, three studies could not show any change in the density of IWMNs in either superficial or deep white matter.^70–72^ A summary of these studies is provided in Table 1. Many studies of altered WMN density in schizophrenia have focused on three key markers, NADPH, MAP2 and NeuN, the latter because it detects all mature neurons.

**Table 1:** Summary of the studies investigating the density of IWMNs underneath the cortex in subjects with schizophrenia

Study	Brain region^a^	Brodmann area^b^	Sample size^c^	Neuronal marker^d^	Density of IWMNs	
					Superficial white matter	Deep white matter
Akbarian, et al.^70^	DLPFC	NR	5:5	NAPDH	Decreased	Increased
Molnar, et al.^73^	DLPFC	NR	18:18	NADPH	Unchanged	Unchanged
Akbarian, et al.^67^	DLPFC	NR	20:20	MAP2	Increased	Unchanged
Anderson, et al.^68^	DLPFC	BA46	5:5	MAP2	Increased	Unchanged
Beasley, et al.^74^	DLPFC	BA9/10	15:15	MAP2	Unchanged	Unchanged
Kirkpatrick, et al.^69^	IPC	BA39	5:7^e^	MAP2	Increased^f^	Not examined
Rioux, et al.^71^	PHG	NR	41:15	MAP2	Unchanged	Increased
Ikeda, et al.^72^	DLPFC	BA9	14:6	NPY	Unchanged	Increased
Eastwood and Harrison^56^	DLPFC	BA22	12:14	NeuN and Reelin	Increased	Unchanged
Eastwood and Harrison^57^	DLPFC	BA22	11:12	NeuN	Increased	Unchanged
Connor, et al.^58^	CWM	BA33	22:45:(15)^g^	NeuN	Increased^g^	Unchanged
Yang, et al.^59^	DLPFC	BA46	37:29	NeuN and SST	Increased	Unchanged
Joshi, et al.^22^	OFC	BA11	38:38	GAD NeuN	Increased	Unchanged
McFadden, et al.^75^	DLPFC	BA9/BA46	39:61	Nissl	Unchanged	Not examined

Adapted from Eastwood and Harrison^56^

^a^*DLPFC* dorsolateral prefrontal cortex, *IPC* inferior parietal cortex, *PHG* parahippocampal gyrus, *CWM* cingulate white matter, *OFC* orbitofrontal cortex

^b^*NR* not reported

^c^Number of subjects with schizophrenia: to the number of control subjects

^d^*NeuN* neuronal nuclear antigen, *MAP2* microtubule associated protein 2, *NADPH* nicotinamide–adenine dinucleotide phosphate-diaphorase, *SST* somatostatin, *NPY* neuropeptide Y

^e^Subjects with schizophrenia split into deficit and nondeficit subgroups

^f^Significant in the deficit subgroup only

^g^Number of cases with bipolar disorder where the increase of IWMN was also observed is shown in brackets

Akbarian, et al.^67^ published the first study showing that subjects with schizophrenia had a significant reduction in the NADPH+ neurons in the superficial white matter adjacent to the cortex in the DLPFC, but in contrast, a significant increase in the density of IWMNs in the deep white matter region.^67^ Molnar, et al.^70^ in the only other study using NADPH as an IWMN marker, observed no significant changes in the density of IWMNs in either the superficial or deep white matter, albeit the groups studied had low sample sizes. This suggested that the use of NADPH as an IWMN marker in studies of their density in schizophrenia may be sensitive to the disease-related increase in IWMN density. Other early studies examined MAP2-positive (MAP2+) IWMNs, and in a second study, Akbarian, et al.^64^ reported that the density of MAP2+ IWMNs was increased in the superficial white matter subjacent to the DLPFC of subjects with schizophrenia. In contrast to their earlier study using NADPH, Akbarian and colleagues did not observe a significant difference in the density of MAP2+ IWMNs in the deep white matter compared to controls.^64^ Increased MAP2+ IWMN density in the superficial white matter was then replicated by Anderson, et al.^65^ in the DLPFC, but they observed no change in the deep white matter.^64^ Interestingly, Kirkpatrick, et al.^66^ showed that schizophrenia subjects with cognitive impairments had a significantly higher density of MAP2+ IWMNs in the superficial white matter subjacent to the inferior parietal cortex (BA39) than either patients with little or no cognitive deficits or healthy controls.^66^ Beasley, et al.^71^ contributed to variability in the reports of IWMN alterations in schizophrenia when they published a conflicting result to Akbarian, et al.^64^ and Anderson, et al.^65^ and could not detect a significant change in MAP2+ IWMN density or spatial distribution between cases with schizophrenia and matched controls, in either the superficial or deep white matter subjacent to the DLPFC. Then, the following year, Rioux, et al.^68^ also showed no change in density of superficial IWMNs when compared to controls when the distribution of MAP2+ IWMNs in the white matter of a different brain region, the parahippocampal gyrus, was examined in subjects with schizophrenia. Since MAP2 is only expressed in a small subset of IWMNs, it is possible that examining MAP2+ neurons, like examining NADPH+ neurons, within the white matter does not provide a true indication of the density of all neurons in the white matter. The breakthrough in the consistency of identifying altered IWMN pathology in schizophrenia came when a series of studies investigating the density of IWMNs was published using antibodies directed against a more global neuronal marker, NeuN. Eastwood and Harrison^53^ provided the first study examining altered IWMN density using immunohistochemical labelling for NeuN and showed that the density of NeuN+ IWMNs was increased in the superficial white matter of the DLPFC. They also showed that some IWMNs express less reelin protein and mRNA^53^; thus reelin expression may identify a subset of IWMNs. The contribution of reelin in the neurogenesis of IWMNs will be discussed later in this review. In a second study, Eastwood and Harrison^54^ replicated this increased density of NeuN+ IWMNs in the DLPFC in a separate cohort of subjects with schizophrenia. Then a few years later, Connor, et al.^55^ showed that the density of NeuN+ IWMNs was increased in the superficial white matter of the cingulate white matter. A couple years after that, further support for increased IWMN density was provided by a larger study from our group Yang, et al.,^56^ where we confirmed that an increased density of NeuN+ IWMNs in the superficial white matter could be found in schizophrenia subjects compared to controls. Yang, et al.^56^ also showed that there was a subset of NeuN+ IWMNs that expressed the neuropeptide SST and that these SST+ IWMNs were also increased in density in the white matter of the DLPFC in subjects with schizophrenia. Interestingly, a proportion of these SST+ neurons also co-expressed NPY and an earlier study by Ikeda, et al.^69^ showed that the density of NPY+ IWMNs was increased in the deep white matter of the DLPFC in people with schizophrenia compared to controls. These studies further suggest that specific subsets of IWMNs might be more altered in the brain in people with schizophrenia. Interestingly, Morris, et al.^73^ demonstrated that *NPY* mRNA, but not *SST mRNA* was significantly reduced in the superficial white matter in those with schizoaffective disorder compared to controls. In a more recent and larger study of the density of IWMNs, Joshi, et al.^19^ showed in a cohort of 38 schizophrenia subjects and matched controls that there was an increase in both GAD+ and NeuN+ IWMNs in the superficial white matter of the orbitofrontal cortex.

These studies that have accessed some of the largest brain tissue cohorts available at the time, provide fairly robust, even though not entirely consistent, evidence for a significant increase in IWMN density subjacent to the cortex in several brain regions implicated in schizophrenia including the frontal, temporal and parietal lobe white matter. These studies also show that NeuN is probably the most reliable immunohistochemical marker for detecting these differences and studies that use only Nissl staining or more differentiated neuronal markers may miss the increased density of IWMNs in schizophrenia. Collectively, these studies reported a generalized increase in the density of neurons in these selected compartments of white matter space^53,54,65,67^ with a possible redistribution towards the deeper white matter.^64,68^ This further suggests that white matter alterations could emerge to be a significant pathology in the development of schizophrenia.

## Underlying mechanisms of white matter neuron pathology in schizophrenia

**Fig. 1:**
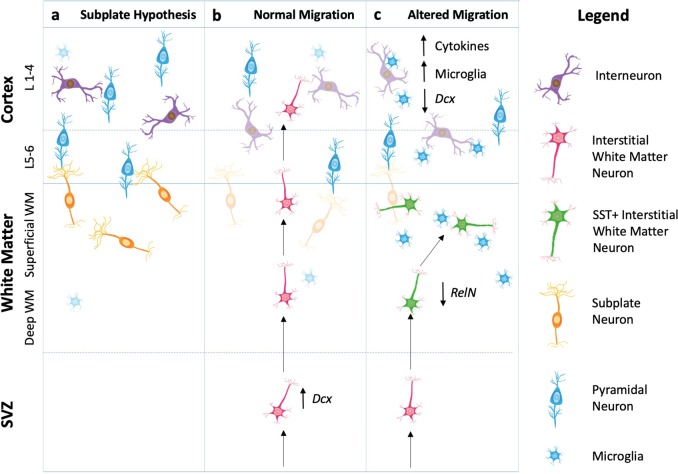
Hypothetical mechanisms for the role of interstitial white matter neurons (IWMNs) in normal and schizophrenia-related brains. a IWMNs may represent remaining subplate neurons that form connections with cortical pyramidal neurons in layers 5-6 of the overlying cortex. **b** IWMNs may be part of a normal restorative brain mechanism, migrating to the cortex in response to particular brain cues. The type of IWMN that responds depends on the brain cue. **c** In schizophrenia, cortical inflammation causes damage to particular neurons (e.g., SST+ interneurons) which may trigger neurogenesis but these SST+ IWMNs fail to migrate radially and terminate in the white matter from early in development

The lack of certainty about the origin of IWMNs has caused some debate in the schizophrenia research community and if resolved could provide a better understanding of the possible biological significance of increased IWMN density in schizophrenia. Early reports about IWMNs mainly focused on the assumption that they are remnants of the subplate, a transient structure beneath the cortical plate in the prenatal and early postnatal brain.^74^ After migration of neurons from the subplate to the developing cortex is completed, it was assumed that most neurons remaining in the subplate undergo apoptosis. However, with the demonstration of a small population of these neurons surviving into adulthood, some believe IWMNs may represent fetal subplate neurons that have escaped apoptosis and subsequently reside in the subcortical white matter of the mature brain that can be readily found in normal human and primate brains (Fig. 1a).^75^ This hypothesis certainly has merit on several grounds. For several decades now, schizophrenia has been considered by some to be a developmental disorder where an insult in utero affects neurodevelopment leading to brain dysfunction in late adolescence/early adulthood.^76^ Furthermore, autism spectrum disorders which have some association with schizophrenia, are also considered to be a neurodevelopmental disorder and there are reports of increased IWMN density underneath the cingulate cortex from post mortem studies of people with autism.^77,78^ Indeed, subplate neurons are at their highest density at birth after which there numbers decline.^61^ It is possible that the environmental insult in utero disturbs the ability of subplate neurons to migrate into the cortical layers leaving them trapped in the white matter. Maternal infection is a risk factor for schizophrenia and in support of this notion we recently showed that prenatal exposure to maternal immune activation particularly in late gestation, resulted in increased SST+ IWMN density in the corpus callosum of the adult rat offspring similar to what is observed in post mortem brains in schizophrenia.^79^ When this increase in density of SST+ IWMNs occurred during development in response to maternal immune activation is still to be determined. As the neurodevelopmental hypothesis has been researched and tested it is clear schizophrenia is linked to multiple environmental risk factors that impact on neurodevelopment from prenatal stages (e.g., maternal infection) right through to adolescence (e.g., cannabis use).^76^ Future studies of multiple environmental risk factors across the developmental timeline should shed light on exactly what can trigger this increase in IWMN density and when it occurs.

An alternative hypothesis suggests that in contrast to subplate neurons, IWMNs could be neurons derived from a distinct neurogenic zone, that is the ventral telencephalon, and may be migrating towards the cortex (Fig. 1b). Since a large proportion of IWMNs are GABAergic, then it is possible that they emanate from the ventral telencephalon or ganglionic eminences during development. Most cortical GABAergic interneurons arise from the medial ganglionic eminence and express PV and SST.^80,81^ On the other hand, approximately 30% of total cortical interneurons are thought to arise from the caudal ganglionic eminences during development and express calretinin, reelin, NPY and vasoactive intestinal peptide.^82–84^ Possibly, GABAergic neurons that do not complete their migration to the cortex per se, reside in the white matter as the IWMNs observed in the normal adult brain. However, the ganglionic eminences are developmental structures making it less likely to be the source of IWMNs detected in adult brains several decades later.

More recent research suggests that possibly a more likely source of these IWMNs is the subependymal zone (SEZ) located along the wall of the lateral ventricle in the adult brain. Indeed, much interest has developed around the discovery that the SEZ is a site of significant neurogenesis in the adult human brain.^85–88^ However, this remains controversial with several reports using mostly immunohistochemical markers suggesting a significant number of neural progenitor cells can be identified in human foetal brain, but this rapidly declines after birth becoming very low or undetectable in adulthood.^86,89,90^ Further evidence to support adult neurogenesis has come from rodent brain injury models (e.g., traumatic brain injury or ischemic brain injury) showing that upregulation of subependymal zone neurogenesis and recruitment of new neurons to the injury site is a physiological response to cortical damage (Reviewed in Ohira^91^). In addition, rodent models show that certain forms of brain injury can upregulate neurogenesis in the subependymal zone, producing new neurons that migrate through the subcortical white matter to the cortex.^91–94^ Neurogenesis in the subependymal zone has also been observed in neurodegenerative diseases of the human brain such as Huntington’s disease, where death of striatal neurons is thought to induce proliferation of neuronal precursors.^95,96^ These observations raise the question ‘Could the cortical pathology in schizophrenia be sufficient enough to trigger a “brain injury” response leading to an increase in neurogenesis in the SEZ?'

A consequence of brain injury is often inflammation. In this regard, there is building evidence for an inflammatory signature in the brain in schizophrenia. There are changes in inflammatory gene expression and immune system pathways in the cortex in schizophrenia including overexpression of pro-inflammatory cytokines.^41,97,98^ There is evidence for infiltration of macrophages into the brain, changes to the blood brain barrier^99^ and possible activation of microglia in the brain in schizophrenia.^100^ In our rat model of maternal immune activation in addition to increased density of SST+ IWMNs, we observed an increase in immunoreactivity for IBA1 (a marker of microglia) in adult rats exposed to this environmental factor.^101^ Further work on this model could determine if maternal infection not only triggers an increase in IWMNs but also leads to a pro-inflammatory state in the brain that persist in adulthood leading to changes in behavior. One limitation of the link between increased IWMN density and neuroinflammation in the development of schizophrenia is that not all cases have a high inflammatory state in their cortex.^97^ Furthermore, in each post mortem study in Table 1 reporting increased IWMN density in schizophrenia, some cases had IWMN densities at or below that for the control groups. What is very interesting though is the findings from Fung, et al.^102^ showing that high levels of inflammatory cytokines including interleukin (IL)-6, IL-1b, IL-8 and SERPINA3 expression in the cortex correlated significantly with higher IWMN density in the OFC in a subset of people with schizophrenia. This suggests that the higher level of cortical inflammation or ‘brain injury’ in some cases of schizophrenia may trigger more neurogenesis in the SEZ generating higher numbers of neurons that in theory should migrate to the overlying cortex and attempt to reverse the cortical pathologies in schizophrenia. However, since the cortical pathologies persist in schizophrenia along with the increased density of IWMNs underneath the damaged cortex, the increased IWMNs is not enough to “repair” cortical damage and additional deficiencies in the development and maturation of IWMN, for example in neuronal migration, also likely play a role in the ongoing neuropathology (Fig. 1c).

For instance, studies of the DLPFC in schizophrenia have identified reduced expression of reelin, a marker of migrating neurons.^12,103,104^ Reelin is a serine protease secreted during development by Cajal-Retzius cells in the marginal zone and is known to guide neuronal migration, positioning and proliferation during corticogenesis.^105–107^ In addition to this, reelin has been shown to have a role in cortical lamination, columnarity and synaptic connectivity.^108,109^ Interestingly, to model decreased reelin expression, Tueting, et al.^110^ developed a heterozygote *reeler* mouse (rl+/−), which showed a variety of morphological, behavioural, and neurochemical changes compared to wild-type mice, that were similar to those observed in people with schizophrenia.^110^ Of importance to this review, Tueting, et al.^110^ observed an increased number of IWMNs under the frontal cortex, suggesting that a partial decrease of reelin expression was enough to produce phenotypic effects like those seen in schizophrenia and may be related to the increased IWMN density in this disorder. We do acknowledge there are a number of animal models that produce similar phenotypes equated to schizophrenia but do not specifically target interneuron markers in their interventions.

More recent evidence that IWMNs may be migrating has come from Fung, et al.^111^ who reported that IWMNs expressed doublecortin mRNA, a marker of immature migrating neurons. Interestingly when the expression of doublecortin in the cortex of schizophrenia cases was low the IWMN density was high underneath the cortex in schizophrenia cases.^111^ Meanwhile, Yang, et al.^56^ observed a number of SST+ IWMNs that did not express NeuN and suggested that these IWMNs may be immature neurons. Once again, the level of cortical SST mRNA negatively correlated with the density of SST+ IWMNs in schizophrenia cases. Some of the GAD-67+ neurons found underneath the cortex also have a morphology consistent with migrating neurons, that is these IWMN neurons have an ovid shape and a leading and trailing process oriented parallel to the pial surface.^19^ These data support the notion of a migration deficit hypothesis in schizophrenia (Fig. 1c).

Taken together, this begs the question, has the increased IWMN density found in the brains of people with schizophrenia been interpreted in the correct way? Whilst it has been traditionally proposed as a possible developmental pathology, it may in fact be representative of a restorative or protective mechanism within the human brain to prevent chronic brain injury. We hypothesize that inflammation is leading to damage within the cortex (as evidenced by a cortical immune signature), and the brain is attempting to mount a repair response via neurogenesis and the recruitment of new neurons from the underlying structures such as the SEZ (evidence of migrating, immature neurons). However, in the schizophrenia perturbed brain, these neurons are not recruited effectively whether that is due to a lack of appropriate molecular and cellular environments or cues, such as alterations to transcription factors being expressed, or abnormal migratory ability of IWMNS leaving them ‘stranded’ in the white matter. For example, *SST* mRNA has been shown to be decreased in the cortex by post mortem tissue studies using in situ hybridization (right PFC^18^ and DLPFC^112^) which is supported by qPCR (DLPFC^28^ and a regional cortical study^113^). However, in the white matter the density of SST+ IWMNs is increased;^56^ perhaps this is a result of attempting to recover this cortical SST interneuron deficit or leading to it.^3^ What could be the consequences for cortical function? Cortical SST+ interneurons are known to inhibit pyramidal neurons in layers II/III and V and PV + interneurons in layer V^114^ thus a reduction in cortical SST expression may result in reduced cortical inhibition allowing pyramidal neuron overexcitation. SST+ interneurons have also been implicated in behaviours such as learning and memory^115^ so it’s possible that changes to cortical SST expression could affect symptoms in people with schizophrenia. What about the SST changes in the white matter, where data suggests that IWMNs are electrically active, and the anatomical and electrophysiological data suggests they are integrated into cortical circuitry, even if only transiently. An intriguing thought is that these IWMNs may be able to influence cortical processing having a detrimental effect leading to some of the brain dysfunction observed in people with schizophrenia? However, the jury is still out on whether there are enough IWMNs to be able to influence total cortical activity.

Another hypothesis is that, as SST is known to be a potent vasoconstrictor, and that SST+ IWMNs are increased in people with schizophrenia, they may be affecting cortical activity by restricting blood flow, which could result in less BOLD activity by fMRI. Characterizing what neurotransmitters are released from IWMNs will be crucial in examining this further. Indeed, complete phenotyping of the IWMN subgroups will be necessary to fully elucidate their biological roles. Future studies should also seek to determine whether the increased density of IWMNs is correlated at all with changes to white matter detected in cases with schizophrenia by DTI, in particular the reduction in white matter volume.^33^ As such IWMNs remain an intriguing cell population, certainly worthy of future research and investigation.
